# Candidate Passive Sensor Suite Technologies for Tactical Combat Casualty Care Environments: Comparative Assessment Study

**DOI:** 10.2196/84900

**Published:** 2026-06-24

**Authors:** Ericka Stoor-Burning, James Gaudaen, Holly Pavliscsak, Jeanette Little

**Affiliations:** 1 United States Army Institute for Surgical Research, Building 1054, Patchel Street, Fort Detrick, MD, 21702, United States, 1 706-993-6862

**Keywords:** autonomous medical documentation, comparative technology performance assessment, military medical documentation, multi-phase assessment methodology, passive medical documentation, tactical combat casualty care, prototype sensor suites, simulation-based assessment

## Abstract

**Background:**

The United States Army Institute for Surgical Research conducted an analysis of 3 prototype sensor suites; all candidates were designed to passively document care delivery in tactical combat casualty care environments.

**Objective:**

This study aims to ensure sensor suites remain resilient and adaptive in complex battlefield environments. This research effort conducts a systematic comparative assessment of prototype solutions.

**Methods:**

The assessment methodology prioritized functionality, usability, and performance. The assessment consisted of three phases: (1) tabletop evaluations, (2) simulated use testing, and (3) a sensor suite rodeo simulation event. The second and third phases included human participants leveraging the technology prototypes in hyperrealistic tactical combat casualty care simulation environments. Additionally, the third phase allowed the researchers to assess the performance of each prototype in a range of operational environmental lighting conditions.

**Results:**

During the tabletop evaluation phase, all 3 prototype sensor suite solutions demonstrated acceptable results (≥1) in the technical specification assessment. The 2-part heuristic analysis revealed variability, where the least complex configurations received the highest assessment scores. To capture and record raw data, scores ranged from 44.6 to 87 on a 100-point scale. To offload and export the raw data, scores ranged from 22.9 to 87.5 on a 100-point scale. During simulated user testing, all 3 sensor suites achieved passing quantitative scores (≥60); the system usability scores (SUS) ranged from 60 to 85 on a 100-point scale. More complex technology configurations received higher usability scores. From a qualitative perspective, vital sign monitor latency display issues led to reliability concerns. All 3 prototypes successfully generated raw data; the individual outputs ranged from 0.06 to 0.13 GB/minute. During the sensor suite rodeo simulation event, all 3 sensor suites achieved passing quantitative scores (≥60); the SUS ranged from 66.7 to 86.7 on a 100-point scale; the most complex technology prototype configuration scored higher. Qualitative findings identified data transfer issues with large file sizes and pairing issues with vital sign monitors. All 3 prototypes successfully generated raw data; the individual outputs varied (ranging from 0.012 to 0.24 GB/min) based on the environmental lighting conditions (full sun, indoor lighting, and low light). However, from a data quality perspective, only 1 camera component produced viable video data in all 3 environments.

**Conclusions:**

The comparative assessment revealed opportunities to combine the strengths of both approaches in a next-generation implementation. This preliminary assessment was constrained by several factors: (1) effective tracking of consumable medical supplies, (2) advancement of artificial intelligence algorithms to process the raw data, and (3) ability to manage multiple casualties or patients. Follow-on evaluations are needed to address these limitations. This systematic, 3-part methodology evaluates early-stage sensor suite prototypes and provides a reproducible framework for advancing battlefield medical technologies.

## Introduction

The Military Health System faces a persistent challenge: while medical documentation is mandated at all echelons [1], providers in high-intensity combat situations must prioritize lifesaving measures over record-keeping, often leading to significant data gaps. Manual, paper-based documentation is not only burdensome but also prone to result-interpretation errors due to varying environmental factors.

To address this challenge, the United States Army Institute of Surgical Research (USAISR), formerly known as the Telemedicine and Advanced Technology Research Center, commenced an effort to prototype systems specifically designed to passively document care delivery within the tactical combat casualty care (TCCC) environment. The sensor suite systems used individual commercial-off-the-shelf components integrated with an edge computing device and/or end user device as shown in Table 1. The 3 specific systems were selected by the government after their respective proposed approaches were determined to have merit.

**Table 1.** Sensor suite prototype configurations.

	Medical Wearable Audio Video Entry	Autonomous Communication Medical Ecosystem	Point of Treatment Aggregator
Audio or video component	PatrolEyes visible or near infrared camera	GoPro Hero 13 Black action camera	MOHOC M2 camera Sennheiser Lapel microphone
Patient vital sign monitor component	Vivalink electrocardiogram patch Vivalink pulse oximeter	Masimo MightySat pulse oximeter	—^a^
Provider activity tracking component	Electromyography armband	Movella DOTs	Movella DOTs
Consumable medical supply use tracking component	—	Near field communication tags	—
Edge computing device	NVIDIA Jetson Orin Nano	—	—
End user device	Samsung Galaxy S23 FE smartphone	Samsung Galaxy S21 smartphone	Dell Latitude 7230 Rugged Extreme tablet

a Not applicable.

Between September 2023 and July 2025, the USAISR conducted a formal analysis of the 3 early-stage prototype sensor suites: the Medical Wearable Audio Video Entry (M-WAVE), the Autonomous Communication Medical Ecosystem (ACME), and the Point of Treatment Aggregator (PoTAg). The results from this initial comparative assessment highlighted opportunities to integrate the unique strengths of each approach, paving the way for a next-generation solution. By identifying complementary features and capabilities among the various prototypes, the assessment established a foundation for designing systems that are resilient and adaptive to complex battlefield environments.

The systematic, 3-part methodology used to evaluate these early-stage sensor suite prototypes provides a robust and reproducible framework for guiding the future development of advanced medical technologies for the battlefield. This approach not only ensures objective comparison and evaluation of devices or systems but also facilitates iterative improvement based on empirical data. Ultimately, such a structured process accelerates the innovation cycle, enhances decision-making for future investments, and supports the creation of integrated solutions that can better address the dynamic and unpredictable needs of battlefield medicine.

The premise behind this prototyping effort is that smart devices, defined as context-aware electronic devices capable of autonomous computation and data exchange [2], offer a unique solution to this TCCC medical documentation capability gap. By integrating these technologies into sensor suites, they serve as a force multiplier for the TCCC level provider, which includes the following:

1. Reduced cognitive load: By automating the capture of patient statuses and caregiver actions, the sensor suite system alleviates the medic’s cognitive burden, allowing them to remain fully “hands-on” with the patient without the distraction of manual documentation [3,4].
2. Data integrity for handoffs: The sensor suite system facilitates the accurate generation of a digital TCCC care, ensuring that life-critical information (including medication dosages and tourniquet times) is seamlessly transferred to the next echelon of care without requiring the medic to pause treatment for manual logging [5].
3. Mass casualty and patient management: Mature sensor suite technologies will enable continuous, simultaneous, and passive monitoring of multiple vital signs across multiple patients through a centralized interface, significantly enhancing a care provider’s situational awareness in complex environments [6].
4. Enhanced operational readiness: Near real-time monitoring of physiological parameters and environmental exposures (eg, noise, radiation, chemical agents) ensures that the health and safety of military members are maintained throughout the mission [7-9].

In the long term, the resulting sensor suite datasets will empower the development of artificial intelligence (AI) and clinical decision support systems to further aid care delivery in austere environments.

This comparative assessment evaluates the performance, usability, and refinement needs of these initial prototypes based on four main hypotheses:

1. Synchronized passive sensor modalities can establish large, reliable TCCC encounter datasets.
2. Passively collected data can effectively train future patient documentation algorithms.
3. Mature algorithms can reliably automate real-time data inputs for digital TCCC cards.
4. Passive, autonomous collection will eliminate the need for manual care provider input at the point of care (POC), improving both efficiency and accuracy.

## Methods

### Overview

**Figure 1.**
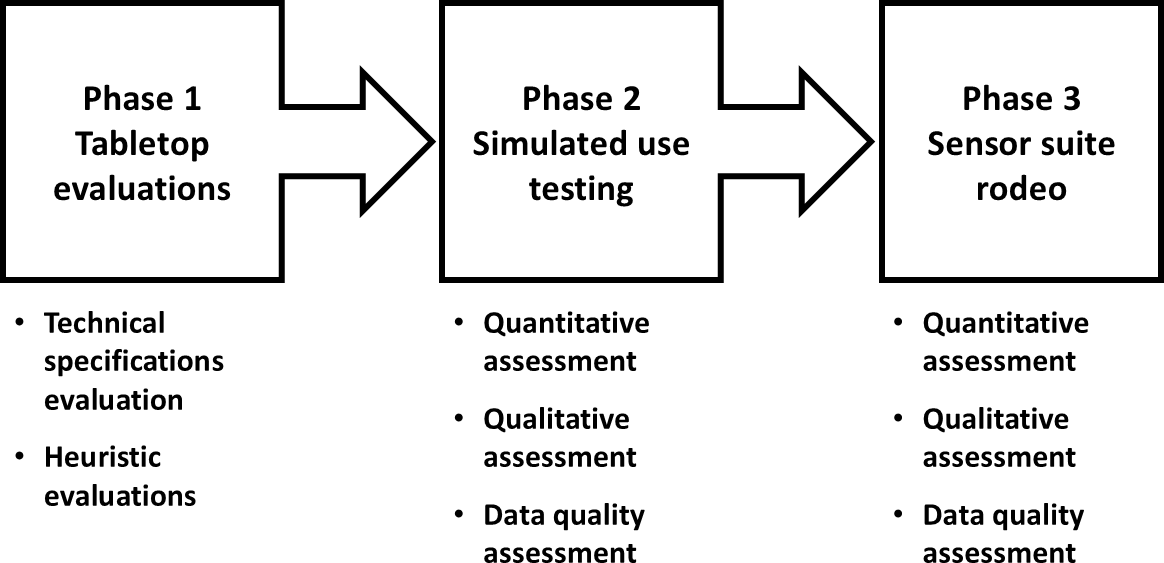
Sensor suite assessment phases.

There were three phases to the assessment: (1) tabletop evaluations, (2) simulated use testing, and (3) a sensor suite rodeo simulation event as shown in Figure 1. The assessment methodology prioritized 3 factors: functionality, usability, and performance. For each prototype, both the entirety of the sensor suite configuration and the individual components were included in the assessment process.

### Study Design or Recruitment

All nonpersonally identifiable information data collected for this assessment were conducted under an exempt research protocol (protocol ID# M-11061). A more expansive, human subject research protocol assessment methodology (protocol ID# M-11057) for the sensor suite prototype assessments has been previously described in the Journal of Medical Internet Research [10].

### Analysis

The sensor suite analysis had 3 discrete phases (Figure 1). The first phase was a benchtop assessment of each component of the individual sensor suites, and the second and third phases required the use of hyper-realistic TCCC simulation scenarios with human subject participants engaging with the sensor suite technologies.

### Phase 1: Tabletop Evaluations

**Figure 2.**
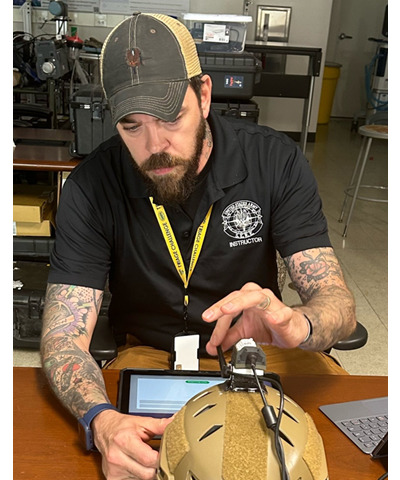
Tabletop evaluations.

The initial phase of the assessment process took place in a controlled laboratory environment and was conducted by independent evaluators systematically reviewing each component of the 3 sensor suite configurations (Figure 2). There were two parts to this tabletop evaluation: (1) a technical specification assessment and (2) a 2-part heuristic analysis.

#### Phase 1A: Technical Specification Assessment

Evaluators considered the overarching sensor suite’s technical specifications and documentation associated with the individual components (Textbox 1). The validity of the technical specifications and documentation was assessed for each defined top-level attribute (Table 2).

Textbox 1. **Sensor suite specifications.**

**Technology**

- Suite includes at least 2 commercial off-the-shelf sensors and a physical data hub
- Commercial off-the-shelf sensors include audio, video, physiological monitors, wearables, and radio frequency identification technologies
- Sensor components are established technologies, not prototypes

- The suite is reliable, effective, and easy to use
- Suite is size, weight, and power efficient, runs for at least 2 hours
- Suite enables passive monitoring of patients, caregivers, and/or resources
- Suite is suitable for collecting combat casualty care data
- Suite is fit for austere, tactical environments

**Data outputs**

- Meets industry standards, is not proprietary
- Data outputs include raw sensor data
- Data separate patient and caregiver data
- All data are downloadable, organized, time-stamped, and include descriptive tags
- Data processing is efficient and effective

**Technical documentation**

- Lists targeted tactical combat casualty care fields
- Details data standards used
- Estimates data output or bandwidth
- Estimates power or battery needs
- Describes software
- Provides user manuals
- Includes both a training and project plan

**Table 2.** Technical specification attributes.

Attribute	Sub attribute
Systemwide factors	Automatic data aggregation Data aggregation system Synchronized data format Time synchronization Data processing steps Key metadata or tags Approximate cost
Form factor	Size (width, length, height) Weight
Device power requirements	Power modality Approximate battery life
Interface	Attaches to (eg, caregiver or patient) Attachment method User interface modality Passive or active collection Required actions (if active)
Data capture and storage	Data type Data format Data streams Data intended use case Key metadata Software required to access data Real-time data availability Time recording modality Sample or frame rate
Device flexibility	Key settings
Data transfer	Wireless transfer capabilities Connection type

Each component of the sensor suite was independently evaluated by 2 separate raters using a 3-point scale (0=lowest, 2=highest). The scores were then consolidated; if the raters provided different scores for the same component, the lower score was recorded as the official score for that component. Next, the scores for all the individual components were averaged.

#### Phase 1B: Heuristic Analysis

A heuristic evaluation is a systematic inspection methodology to quantify the usability of the sensor suite prototype interface design. During this phase of the analysis, 2 subject matter expert evaluators examined the interface and determined compliance with recognized usability principles, specifically the Nielsen Norman Group 10 Usability Heuristics model [7], as outlined in Table 3.

**Table 3.** Nielsen Norman Group’s 10 heuristics.

Heuristic	Example
Visibility of system status	Keeps users informed with timely feedback.
Match between system and real world	Leverages end user language and real-world conventions; avoids jargon and presents information logically.
User control and freedom	Provides clear exits so users can easily undo mistakes.
Consistency and standards	Ensures consistency by following platform and industry conventions.
Error prevention	Prevents problems before they occur; eliminates error-prone conditions or confirms risky actions.
Recognition rather than recall	Reduces memory load by making key elements and information visible or easily accessible.
Flexibility and efficiency of use	Provides shortcuts for experienced users and allows customization of frequent actions.
Esthetic and minimalist design	Removes irrelevant or rarely needed information from the interface.
Help users recognize, diagnose, and record from error	Writes error messages in plain language, clearly stating the problem and solution.
Help and documentation	Aims for a self-explanatory system but provides helpful documentation if needed.

There were 2 discrete parts to the heuristic assessment. Task 1 focused on the ability of the sensor suite components to capture and record data in a usable fashion (eg, openly accessible, and not using proprietary data formats). Task 2 focused on the ability to offload and leverage the data that were generated by the sensor suite components. To conduct the task 1 heuristic assessment, all the sensor suite components were powered up, connectivity was established in accordance with the performer’s documentation, and data collection or recording was commenced. Once this process was completed, the data offloaded from the sensor suites were exported to a secure, cloud-based research data repository (eg, the task 2 heuristic assessment). In instances where the data were not generated by the individual sensor component in task 1, the task 2 heuristic assessment did not occur.

For each individual sensor suite component, it was assessed across 10 discrete heuristic factors, described in Table 3, with a maximum score of 2 per factor resulting in a maximum of 20 total points. Evaluator scores were averaged to determine a final score. Each sensor suite configuration score was then calculated using the sum of the individual components.

The resulting summary score was then divided by the total possible points a sensor suite could receive, which was based on the total number of component devices multiplied by 20 (eg, number of components × 20). After this division, the resulting number was multiplied by 100 to standardize the 100-point scale, as follows:


$$\text{Sensor suite configuration score}=\left(\frac{s}{n\times 20}\right)\times 100$$

where *s* is the sum of all individual suite component heuristic scores and *n* is the total number of individual sensor suite component devices.

This scoring process was used for both the task 1 and task 2 heuristic assessments. For the purposes of this assessment, a total score of 60 or higher was considered a passing score, a standard used with other validated usability instruments. In addition to providing numerical scores, each evaluator added comments to their ratings. These qualitative comments were combined into a thematic analysis for each of the heuristics to add more information about the quantitative ratings.

### Phase 2: Simulated Use Testing

**Figure 3.**
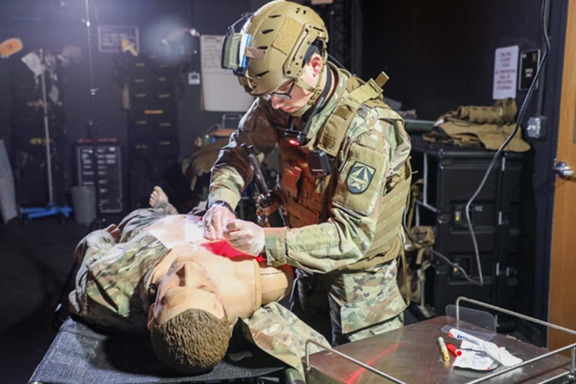
Simulated use testing.

Simulated use cases involving end user testing, and data quality assessments were conducted as a second phase of the sensor suite evaluation [11] (Figure 3). The simulated use cases were carried out in a controlled field setting, where the prototypes were deployed under carefully managed conditions and known reference points for comparison (Textbox 2).

Textbox 2. **Simulated use testing live actor scenarios.**

**Clinical presentations**

- Open tibia or fibula fracture
- Minor head laceration
- Second degree burn to upper extremity

**Expected treatments**

- Splinting of leg
- Management of minor laceration
- Burn management
- Initiating intravenous
- Administering medications
- Hypothermia prevention

During the simulated use testing phase of the assessment, each sensor suite prototype was used within a high-fidelity simulation where the care provider or medic participant was a TCCC provider (eg, tier 1, 2, and/or 3). The care provider or medic participant performed casualty care on live actors. The simulation team used testing scenarios that were run twice to ensure that a complete dataset was collected during the assessment. Once each simulation was complete, the participant was given two surveys to fill out: a quantitative instrument and a qualitative tool.

#### Phase 2A: Quantitative Assessment

The objective, quantitative instrument selected for this assessment was the System Usability Scale (SUS) [12] questionnaire. The SUS instrument is a validated, standardized survey that has been used across all types of systems and technologies to evaluate usability. Once the final SUS scores are calculated, they are stratified against published norms based on the technology types. In general, a total SUS score of 60 or higher is considered passing from a usability perspective, and this widely accepted criterion was used for the sensor suite evaluation.

#### Phase 2B: Qualitative Assessment

The qualitative assessment consisted of specific short answer questions that more specifically targeted the sensor suites that are being assessed in a subjective manner. This assessment was limited in scope to the sensor suite components that were worn by the care provider or medic (eg, audio or video and activity tracking sensor components) during the simulated use case assessment.

#### Phase 2C: Data Quality Assessment

The data quality assessment was conducted by 2 evaluators after the sensor suite data were extracted from simulated use testing events. The primary focus of this assessment was to ascertain the usefulness of the data outputs for algorithms to achieve passive, autonomous documentation based on the defined data output specifications (Textbox 1). While the preponderance of this assessment was qualitative and presented in a narrative format, the overall data output quality from each sensor suite configuration was also quantitatively rated. This rating used a 3-point scale, where a “2” indicated that the data quality satisfied the requirements, a “1” meant the data quality required additional clarification to determine minimal viability, and a “0” signified that the data quality did not meet the minimum viability required.

The scoring process was designed to minimize the risk of overestimating performance or outcomes. By employing stringent criteria and favoring lower or more cautious scores when uncertainty existed, the process ensured that only clear evidence of achievement or competency was rewarded with higher marks. Ambiguous, incomplete, or borderline cases were typically scored on the lower end of the scale, reducing the likelihood of inflating results. Additionally, the use of standardized rubrics and, where applicable, multiple reviewers further contributed to the conservative nature by encouraging consistency and discouraging leniency. This approach ultimately provided a more reliable and defensible assessment, emphasizing accuracy and restraint over optimism or assumption.

### Phase 3: Sensor Suite Rodeo Simulation Event

**Figure 4.**
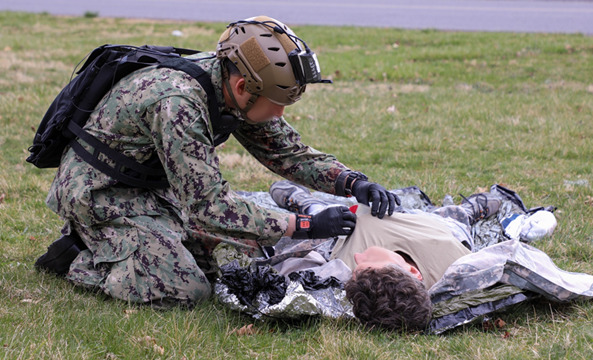
Sensor suite rodeo simulation event.

To directly compare each sensor suite configuration, the team conducted a “sensor suite rodeo” simulation event in March 2025 (Figure 4). The rodeo event was conducted using 3 different environmental venues (outdoor or full sun, indoor or full lighting, and low-light conditions). The assessment team collected data within each setting, which had 9 discrete clinical TCCC skills [13] using both live actions and manikins (Table 4). End user feedback was collected after each simulation through the same quantitative and qualitative survey instruments used in the simulated use assessment (eg, phase 2).

**Table 4.** Sensor suite rodeo simulation event summary.

Lighting conditions	Clinical TCCC^a^ skills performed	Casualty type
Outdoor or full sun	Chest needle decompression Apply pressure dressing to open extremity fracture Apply chest seal	Manikin Live actor with moulage Live actor with moulage
Indoor full lighting	Nasopharyngeal airway placement Start intravenous line Medication administration	Manikin Live actor (no moulage) Live actor with moulage
Low-light conditions	Cricothyrotomy Tourniquet placement Pelvic binder placement	Manikin Live actor with moulage Live actor without moulage

a TCCC: tactical combat casualty care.

### Ethical Considerations

#### Institutional Review Board or Ethics Board Approval

This protocol was reviewed and approved by the United States Army Medical Research and Development Command Institutional Review Board Office, which included human participants’ ethics review. The approved protocol (#M-11057) was classified as minimal risk to human participants and undergoes annual continuing reviews by the Institutional Review Board Office to ensure the safety and ethical considerations of research on all human participants in our research.

#### Informed Consent

Prior to conducting simulation scenarios, individual participants were provided with a consent form. This form explained the activities, potential risks, and data collection that the principal investigator would like to conduct. If the participant agrees to participate, they are asked to sign the consent form, either in hard copy or electronically.

#### Privacy and Confidentiality

All simulation participants (eg, care providers and simulated patients) are assigned a participant ID to be associated with collected demographics, video, and physiological signal recordings. All data are stored in Department of War (DoW)–compliant and Health Insurance Portability and Accountability Act–compliant safety and security compliant e-storage facilities. All hard copy documents, including completed consent forms, are stored in locked filing cabinets at the USAISR. Following data collection sessions, all recordings are checked to ensure that any nonconsented individual’s identifiable information was not recorded. If any inadvertent recordings were made, the principal investigator or a study team member deleted these segments of recordings. All recordings are stored securely in DoW-approved, USAISR-maintained server or repository, including a network attached storage at Fort Detrick, Maryland. Additionally, photo release waivers for image use in nonresearch activities also accompany the consent forms at the start of participation but are entirely optional for participation in the study.

#### Compensation Details

Military volunteers who meet the eligibility requirements and consent to participate in the study had the opportunity to participate in volunteer hours as a participation incentive. After completion of their participation in the study, the participant may request a letter of participation from the research team. The eligibility for volunteer hours toward service recognition will be determined by the units or offices who grant the volunteer hours. The research team will provide the letter of participation in an electronic format to the participant’s email address. This process will be mirrored at all data collection locations. There are no monetary incentives or compensation for this research effort for volunteers.

## Results

During the first phase of the assessment (eg, the tabletop evaluations), all 3 prototype sensor suite solutions demonstrated acceptable results (scores >1) during the technical specification assessment. The ACME and PoTAg sensor suites received the maximum possible scores for specific technical specifications. The ACME configuration stood out for its intuitive form factor, and the PoTAg prototype received distinction for its power requirements, data capture and storage, and device flexibility, as noted in Table 5.

**Table 5.** Results of sensor suite system wide technical specifications assessment^a^.

Technical specification	M-WAVE^b^	ACME^c^	PoTAg^d^
	Average score	Average score	Average score
Form factor	1.7	2.0	1.8
Power requirements	1.7	1.8	2
Interface	1.50	1.6	1.3
Data capture and storage	1.8	1.6	2
Device flexibility	1.8	1.8	2
Data transfer	1.8	1.8	1.8

a The evaluation used a 3-point scale (0=lowest and 2=highest) for scoring each attribute where a score of >1 is considered acceptable or passing. Two research team evaluators conducted this tabletop assessment separately; their individual scores were averaged.

b M-WAVE: Medical Wearable Audio Video Entry.

c ACME: Autonomous Communication Medical Ecosystem.

d PoTAg: Point of Treatment Aggregator.

The results of the 2-part, tabletop-based heuristics analysis revealed variability, where the least complex configuration (ACME) received the highest assessment scores from the research team (Table 6). To capture and record raw data, the quantitative assessment results ranged from 44.6 (M-WAVE) to 87 (ACME) on a 100-point scale. To offload and export the raw data, the scoring ranged from 22.9 (M-WAVE) to 87.5 (ACME) on a 100-point scale.

**Table 6.** Results of heuristic analysis^a^.

Heuristic analysis criteria	M-WAVE^b^	ACME^c^	PoTAg^d^
Task 1: Capture or record data score	44.9	87	67.5
Task 2: Offload or export data score	22.9	87.5	75

a Due to the varied number of sensor suite components, scores were adjusted to a 100-point scale (0=lowest and 100=highest). Two research team evaluators conducted this tabletop assessment separately; their individual scores were averaged.

b M-WAVE: Medical Wearable Audio Video Entry.

c ACME: Autonomous Communication Medical Ecosystem.

d PoTAg: Point of Treatment Aggregator.

During the second phase of the assessment (eg, simulated use testing), the quantitative assessment portion resulted in SUS scores for each sensor suite prototype. The M-WAVE configuration total score was 85, the ACME SUS score was 60, and the PoTAg total score was 80. While all 3 suites received passing scores (>60), it is noteworthy that the M-WAVE prototype, which had the lowest phase 1 assessment scores, received the highest usability scores from the TCCC provider end users.

During simulated user testing, all 3 sensor suites achieved passing quantitative scores (>60); the system usability scores (SUS) ranged from 60 to 85 on a 100-point scale. The more complex technology configurations (M-WAVE and PoTAg) received higher usability scores from the TCCC provider end users.

The qualitative assessment inputs from the simulated use testing were organized thematically by sensor suite prototype, as shown in Textbox 3. M-WAVE vital sign monitor latency display issues led to reliability concerns. Additionally, the NFC tags associated with the ACME prototype required active engagement by the TCCC provider, which did not align with the overarching passive data collection objective.

Textbox 3. **Simulated use testing: qualitative thematic analysis. Thematic qualitative input was provided by 2 tactical combat casualty care (TCCC) providers.**

**Medical Wearable Audio Video Entry**

- Participants found that the use of the Medical Wearable Audio Video Entry sensor suite did not encumber the care delivery process.
- One participant noted the bulkiness of the MindRove electromyography armband component, specifically that it would make it hard to roll up their operational camouflage pattern sleeves when required.
- With respect to the Vivalink Pulse Oximeter, it was noted that there was a delay in displaying the patient vital signs and pairing with the rest of the sensor suite system.

**Autonomous Communication Medical Ecosystem**

- Participants found the use of the Autonomous Communication Medical Ecosystem sensor suite was easy to use.
- Participants specifically noted that the near-field communication tags interfered with the care delivery workflow and were cumbersome to use.
- One participant stated they failed to scan the near-field communication tags in more than one instance during the simulation events.

**Point of Treatment Aggregator**

- The MOHOC M2 camera and Sennheiser Lapel microphone were unobtrusive, and the hard-wired connections from these components did not get in the way of the care provider or medic actions.
- Participants found that the sensor suite did not interfere with care delivery progress.

The data quality assessment portion revealed that all 3 prototypes successfully generated raw data outputs in file formats that were compliant with the sensor suite specifications (Textbox 1). However, the raw data generated by the 3 sensor suite configurations varied significantly in terms of gigabytes/minute (Table 7). The M-WAVE configuration generated 0.06 GB/minute of raw data outputs, the PoTAg prototype generated 0.1 GB/minute, and the ACME sensor suite generated 0.13 GB/minute. In disconnected, denied, intermittent, and limited operational environments, where synchronous cloud-based connectivity is not an option, the ability to locally process the raw sensor data efficiently is a priority, so maximum data efficiencies are desired (eg, lower amounts of data per minute). The M-WAVE sensor suite prototype demonstrated the most promise, but all 3 prototypes will require additional refinement to streamline the raw data outputs to rates that are more suitable for processing by local edge compute devices in TCCC environments in the future.

**Table 7.** Simulated use testing: data file sizes.

Sensor suite	GB/min
M-WAVE^a^	0.06
ACME^b^	0.13
PoTAg^c^	0.10

a M-WAVE: medical wearable audio video entry.

b ACME: autonomous communication medical ecosystem.

c PoTAg: point of treatment aggregator.

Additionally, the evaluation team noted specific quality constraints about the data generated by all 3 initial prototypes (Textbox 4). The M-WAVE camera had the most flexibility in terms of lighting conditions, but the mounting on the TCCC provider had to be refined to a helmet-mounted position from the chest to ensure appropriate field of view. The Movella Dot IMU sensors leveraged by both the ACME and PoTAg prototypes generated concerns about data file sizes and reliability. Finally, the cabling associated with the PoTAg microphone was prone to disconnection, making data outputs unreliable.

Textbox 4. **Simulated use testing—data quality assessment: qualitative thematic analysis. Thematic qualitative input was provided by 2 members of the research team.**

**Medical Wearable Audio Video Entry**

- The PatrolEyes visible or near-infrared camera automatically transitions between the modes based on lighting conditions (visible vs near-infrared).
- When the camera was affixed to the chest (as intended by the performer), it provided a narrow field of view that did not align with the care provider or medic’s line of sight, which impacts the quality of the video data generated, which in turn could impact future algorithm detection performance.

**Autonomous Communication Medical Ecosystem**

- The right and left Movella Dot inertial measurement unit (IMU) sensor data are not collected simultaneously into a single data output file as expected; instead, the system generates 2 separate data (.csv format) files. As a result, it is possible to have a slight data differential (milliseconds) between the right and left IMU data files during data collection. The origins of this potential data discrepancy would need to be further explored in future assessments to fully appreciate the impact.

**Point of Treatment Aggregator**

- During the tactical combat casualty care simulations, one of the Movella Dot sensor components consistently disconnected, and as a result, no IMU activity data for that sensor were generated during that data collection event.
- In one collection event, the hard-wired audio connection from the microphone to the end user device disconnected, resulting in a loss of data.

The third phase of the assessment (eg, sensor suite rodeo simulation assessment) resulted in quantitative, qualitative, and data quality assessments. The sensor suite rodeo was the first opportunity to assess the performance of each prototype sensor suite in a range of operational environmental conditions, rather than a fixed laboratory environment.

The quantitative assessment portion resulted in separate SUS calculated scores for each sensor suite prototype, with all receiving passing scores (>60). M-WAVE had a total SUS score of 86.67. ACME’s SUS score was 66.67, and the PoTAg received a score of 76.67.

The SUS scores ranged from 66.7 to 86.7 on a 100-point scale. The M-WAVE sensor suite prototype received the highest usability scores from the TCCC provider end users, mirroring the results from the phase 2 simulated use testing.

The qualitative assessment inputs of the sensor suite rodeo simulated use testing were organized thematically by sensor suite prototype and further stratified based on simulation participant (TCCC provider) and researcher inputs as shown in (Textboxes5  6), respectively. From the simulation participant perspective, the M-WAVE prototype was noted to have latency issues with its vital sign monitor component, and the electromyography armband required acclimation by the simulation end users.

Textbox 5. **Sensor suite rodeo simulation qualitative assessment: simulation participant thematic analysis. Thematic qualitative input was provided by 3 simulation participant evaluators or tactical combat casualty care (TCCC) providers.**

**Medical Wearable Audio Video Entry**

- Medical Wearable Audio Video Entry sensor suite was easy to use and did not hinder care
- Vivalink SpO2 data latency on reduced confidence in accuracy

- MindRove electromyography armband and NVIDIA edge computing device felt bulky at first but were not distracting during use

**Autonomous Communication Medical Ecosystem**

- Autonomous Communication Medical Ecosystem sensor suite was easy to use and did not hinder care

- GoPro and Masimo interfaces were familiar to users

**Point of Treatment Aggregator**

- Point of Treatment Aggregator sensor suite was easy to use and did not hinder care
- Audio or video components and wired connections did not interfere with care

Textbox 6. **Sensor suite rodeo simulation qualitative assessment: researcher thematic analysis. Thematic qualitative input was provided by 4 research team members.**

**Medical Wearable Audio Video Entry**

- Medical Wearable Audio Video Entry (M-WAVE) setup is cumbersome and requires components to sync in a specific order
- The M-WAVE app has limited functions, lacks troubleshooting options, and does not clearly show setup status
- Large video files do not auto-transfer, and M-WAVE does not notify users of failed transfers
- M-WAVE file naming makes offloading multiple events tedious and risks data errors
- Offloading less than 5 GB of data from the edge computing device requires third-party software

**Autonomous Communication Medical Ecosystem**

- Autonomous Communication Medical Ecosystem app was simple and showed clear component statuses
- Movella Dot inertial measurement unit setup with end user device was streamlined

- Pairing Masimo sensor required toggling Bluetooth, or vital sign monitor data were lost

**Point of Treatment Aggregator**

- No setup issues with the Point of Treatment Aggregator were noted by the research team
- Sennheiser microphone mini-jack connection to the tablet was unreliable due to its proprietary plug

The researchers conducting assessments of the prototypes noted concerns with the complexity and reliability of the raw data transfers with the M-WAVE prototype, pairing issues with the ACME components, and the vulnerability of hardwired connections on the PoTAg configurations (Textbox 6).

**Figure 5.**
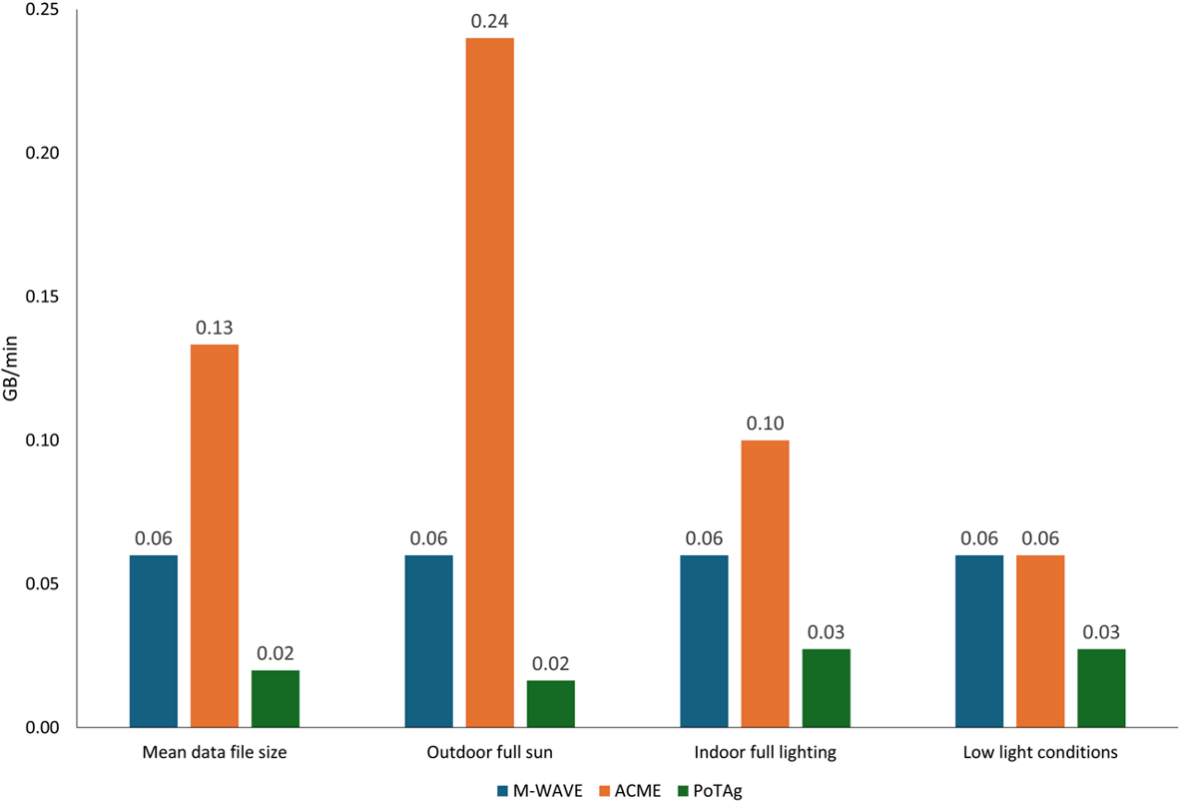
Sensor suite rodeo simulation: data file sizes (GB/min). ACME: Autonomous Communication Medical Ecosystem; M-WAVE: Medical Wearable Audio Video Entry; PoTAg: Point of Treatment Aggregator.

With respect to the data quality assessment, all 3 sensor suite prototypes were able to generate raw data outputs successfully during the sensor suite rodeo simulation event. The sensor suite data outputs varied (ranging from 0.012 to 0.24 GB/min) based on the environmental lighting conditions (full sun, indoor lighting, and low light), as shown in Figure 5. The M-WAVE prototype generated data at consistent rates regardless of lighting conditions. The PoTAg configuration provided the lowest (most desirable) raw data outputs, even with variability in different lighting conditions. However, from a data quality perspective, only 1 camera component, the visible or near infrared (NIR) camera leveraged by the M-WAVE configuration produced viable video data in all 3 environments.

The evaluation team noted specific data quality constraints, which are highlighted in Textbox 7. The camera components leveraged by the ACME and PoTAg prototypes were not optimized for all lighting conditions, whereas the NIR or visible camera leveraged by the M-WAVE solution was ideally suited for all conditions that were assessed in this phase.

Textbox 7. **Sensor suite rodeo: data quality assessment: qualitative thematic analysis. Thematic qualitative input was provided by 2 research team members.**

**Medical Wearable Audio Video Entry**

- Helmet mounting improved video field of view and made camera adjustment easier. A preview feature before recording is recommended
- Medical Wearable Audio Video Entry video was consistently clear in all lighting conditions

**Autonomous Communication Medical Ecosystem**

- GoPro Hero 13 had poor video quality in low light due to reduced visibility

**Point of Treatment Aggregator**

- MOHOC M2 video was washed out in bright sunlight outdoors
- Sennheiser microphones’ wired connection caused unreliable and incomplete audio data

Each of the 3 sensor suite systems assessed during these 3 phased evaluation processes had unique solutions with varying levels of complexity and performance, which directly impacted the results (Table 8). None of the sensor suites were fully optimized at this early stage of prototype development, and the systematic assessment of each configuration identified opportunities for improvement in future phases.

**Table 8.** Summary of sensor suite prototype strengths and weaknesses.

	M-WAVE^a^	ACME^b^	PoTAg^c^
Strengths	PatrolEyes visible or NIR camera auto-switched modes MindRove electromyography^d^ armband outperformed other IMUs^f^ ECD^g^ and EUD^h^ worked well with files less than 1 GB Sensor suite data were consistent in all conditions	Simple setup VSM^e^ component was optimized Only prototype tracking resource use	Reliable prototype Data were efficient but varied slightly by environment
Weaknesses	No resource usage component Vivalink ECG^i^ Patch and Pulse Oximeter were hard to configure and had latency issues ECD and EUD could not manage files less than 1 GB	NFC^j^ resource tracking was poorly received by users and was not passive Camera struggled in low light conditions Raw data generation was inefficient and inconsistent across environments	Limited scope; lacked both VSM and resource usage component The camera did not perform optimally in bright sunlight, impacting data quality The directional nature of the Sennheiser Lapel microphone limited audio capture Specific components required hard-wired connections to the EUD, making it vulnerable to disruptions during use

a M-WAVE: Medical Wearable Audio Video Entry.

b ACME: Autonomous Communication Medical Ecosystem.

c PoTAg: Point of Treatment Aggregator.

d EMG: electromyography.

e VSM: vital sign monitor.

f IMU: inertial measurement unit.

g ECD: edge computing device.

h EUD: end user device.

i ECG: electrocardiogram.

j NFC: near field communication.

## Discussion

### Principal Findings

This 3-part methodology (Figure 1) demonstrated the value of employing a systematic methodology for technology assessments, including, but not limited to, future advanced development of sensor suite prototypes. This discovery-based research approach, including tabletop exercises and systematic technological evaluations, serves as a critical precursor to more formal, controlled studies; it narrows down the vast field of possibilities to a manageable number of promising avenues for further investigation.

While the resulting data from this initial assessment do not yield large enough sample sizes to be assessed for statistical significance, the mixed methods approach is repeatable from both a quantitative and qualitative perspective and can be implemented at a larger scale for more mature technology prototypes.

There are several limitations to the findings of this initial, early-stage prototype assessment. These constraints are expected at this early stage of the prototype development process, as the continuum of technology refinements is intended to be a multiphase, Agile development process [14].

The first limitation was the lack of a prototype solution that was effective in providing passive tracking of the use of consumable medical supplies at the POC. This is an unmet need based on the results of this initial assessment. Future solutions may be dependent upon computer vision algorithms, where tactical cameras can detect consumable medical supplies (eg, tourniquets, blood products) using AI resources, but this assumption will need to be tested and proven in the future phases.

A second confounder is that the data quality assessments performed during this initial assessment were constrained to raw data outputs from the individual components. The initial raw data outputs will require efficiencies to be of future value. Furthermore, providing a complete data assessment requires a systematic evaluation of the edge computing component’s ability to process AI algorithms in disconnected, denied, intermittent, and limited environments and generate orchestrated and consolidated data outputs. This could not be addressed in this evaluation because the AI algorithms were under development. Future, final data outputs will also need to be assessed for their potential to be exported to patient record systems (eg, Battlefield Assisted Trauma Distributed Observation Kit, Operational Medicine Information Systems–Army) and Command and Control systems (eg, MAVIN smart system et al.) in an interoperable fashion to achieve the overarching, autonomous documentation objectives.

Additionally, the results of this assessment are limited with respect to the sensor suite prototypes’ abilities to manage multiple patients or casualties at the POC in mass casualty environments.

Finally, mature sensor suite systems will need to be hardened for the operational environment (eg, ruggedized) and will consider the constraints of cyber and electronic warfare risks. Cyber and electronic warfare protections ensure that future sensor suite technologies for use in TCCC environments do not place service members on the battlefield at risk by generating electronic signatures that can be detected by adversaries. Follow-on development and cyber-specific assessments will be required to address these issues.

### Conclusions

The systematic, 3-part methodology leveraged to evaluate the initial, early-stage sensor suite prototypes is a reproducible process to determine the direction of advanced battlefield medical technology capabilities. The outcomes of this initial comparative assessment revealed opportunities to blend the strengths of each sensor suite prototype approach into a next-generation implementation.

Based on the quantitative results of the tabletop assessment, the form factor of the ACME prototype and the size, weight, and power and data management approaches leveraged by the PoTAg solution should be modeled in future research. The quantitative data from the phase 2 and 3 assessments demonstrate that the M-WAVE configuration was favored by the end users with respect to overall system usability. The raw data output measurements revealed that the M-WAVE provided the most consistent data in all environments, but the PoTAg provided the greatest data efficiencies. All thematic, quantitative data will be leveraged to drive the next generation of sensor suite prototypes.

Further refinement of the prototype sensor suites will adopt a best-of-breed collaborative approach, leveraging the superior components identified in this assessment from both a quantitative and qualitative perspective into a next-generation unified system. There are several notable commercial-off-the-shelf components recommended to be advanced as best-of-breed candidates: the PatrolEyes visible or NIR camera, the Masimo Pulse Oximeter, and the MindRove EMG armband.

This systematic evaluation process is a critical step in determining the shape and direction of future medical capabilities on the battlefield. Technologies inserted into military medicine must improve battlefield health care, making it more effective without compromising the quality of care. To that end, the Institute of Surgical Research is committed to providing fair and unbiased assessments of prototype capabilities, which align with key Defense Health Agency and DoW tenets.

## Abbreviations

ACME: Autonomous Communication Medical Ecosystem
AI: artificial intelligence
DoW: Department of War
M-WAVE: Medical Wearable Audio Video Entry
NIR: near infrared
POC: point of care
PoTAg: Point of Treatment Aggregator
SUS: system usability score
TCCC: Tactical Combat Casualty Care
USAISR: United States Army Institute for Surgical Research
